# Non‐erythropoietic erythropoietin‐derived peptide protects mice from systemic lupus erythematosus

**DOI:** 10.1111/jcmm.13608

**Published:** 2018-03-23

**Authors:** Bo Huang, Juntao Jiang, Bangwei Luo, Wen Zhu, Yuqi Liu, Zhishang Wang, Zhiren Zhang

**Affiliations:** ^1^ Institute of Immunology Army Medical University Chongqing China

**Keywords:** erythropoietin, inflammation, macrophage, systemic lupus erythematosus

## Abstract

Systemic lupus erythematosus (SLE) is an autoimmune disease, which results in various organ pathologies. However, current treatment towards SLE is suboptimal. Erythropoietin (EPO) has been shown to promote SLE recovery, but clinical application can be limited by its haematopoiesis‐stimulating effects. EPO‐derived helix‐B peptide (ARA290) is non‐erythrogenic but has been reported to retain the anti‐inflammatory and tissue‐protective functions of EPO. Therefore, here we investigated the effects and potential mechanisms of ARA290 on SLE. The administration of ARA290 to pristane‐induced SLE and MRL/*lpr* mice significantly suppressed the level of serum antinuclear autoantibodies (ANAs) and anti‐dsDNA autoantibodies, reduced the deposition of IgG and C3, and ameliorated the nephritis symptoms. Moreover, the serum concentrations of inflammatory cytokine IL‐6, MCP‐1 and TNF‐α in SLE mice were reduced by ARA290. Further, ARA290 decreased the number of apoptotic cells in kidney. In vitro experiment revealed that ARA290 inhibited the inflammatory activation of macrophages and promoted the phagocytotic function of macrophages to apoptotic cells. Finally, ARA290 did not induce haematopoiesis during treatment. In conclusion, ARA290 ameliorated SLE, which at least could be partly due to its anti‐inflammatory and apoptotic cell clearance promoting effects, without stimulating haematopoiesis, suggesting that ARA290 could be a hopeful candidate for SLE treatment.

## 1 INTRODUCTION

Systemic lupus erythematosus (SLE) is a persistent growing global disease, whose incidence has almost increased 25% between 2003 and 2008 in the developed country.^1^ As a chronic multisystem autoimmune disease, SLE can potentially lead to serious organ complications, such as massive lymphadenopathy splenomegaly, among which lupus nephritis is the most important cause of death.^2^ However, recent progress in the treatment of SLE has been rare, the only new therapy approved for SLE during the last 50 years is belimumab, suggesting that effort should be more focused on exploring new therapies.^3^

SLE is a multifarious and chronic inflammatory autoimmune disorder with a wide range of clinical symptoms.^4^, ^5^ In patients with SLE, the immune system is subverted to against self‐antigens and the consequent persistent inflammatory response elicits a vicious cycle of immune‐cell activation and tissue damage.^6^ The deregulated production of pro‐inflammatory cytokines, such as TNF‐α and IL‐6, play a key role in immune dysfunction, mediating tissue inflammation and organ damage in SLE.^7^ TNF‐α levels were found to increase in patients with SLE and were associated with the disease activity of SLE. The blocking of TNF‐α function in patients with SLE decreased the disease severity.^8^, ^9^ In addition, in humans and mice with lupus nephritis, IL‐6 promoted autoantibody production and was correlated with the SLE activity.^10^, ^11^ Therefore, the restrain of the inflammatory response could facilitate the improvement of SLE.

While much of the pathophysiology and therapy of SLE has focused on autoimmune B and T cells of the adaptive immune system, recently, phenotypic and functional abnormalities in macrophages are increasingly recognized to play a role in this complex disease. Accumulated evidences suggested a more active role of macrophages in mediating tissue inflammation and injury in SLE, including high inflammatory cytokine production and defective phagocytic capacity. In lupus, macrophages are recognized as a major source of the inflammatory cytokines, which may promote the activation of autoreactive T and B cells. Moreover, macrophages in lupus display a defective phagocytic function, enabling the aberrant accumulation of apoptotic debris, which in turn can lead to a sequel of autoimmune events. In healthy individuals, macrophages remove dead and dying cells in an immune‐silent way. The rapid elimination of apoptotic cells by phagocytes can prevent cell membrane rupture and the leakage of cell contents, to avoid the so‐called secondary necrosis of apoptotic cells.^12^, ^13^ Following the phagocytosis of apoptotic cells, macrophages can produce and release anti‐inflammatory cytokines, such as TGF‐β,^14^ which play an immune regulatory role, and avoid the activation of the immune response to autoantigens and autoimmunity. However, in SLE, the ability of apoptotic cell clearance is deficient, leading to the accumulation of the apoptotic cells, resulting to the secondary necrosis of apoptotic cells and the subsequent activation of self‐immune response.^15^ Therefore, the regulation of macrophage function plays an important role in maintaining homeostasis and inhibiting the occurrence of autoimmune diseases.

Erythropoietin (EPO) is a larvaceous cytoprotective glycoprotein that was initially identified as an essential growth factor of haemopoiesis through the homodimer EPO receptor (EPOR_2_) and widely applied to treat the anaemia.^16^ However, EPORs were found to be expressed in macrophages,^17^, ^18^, ^19^ and EPO signalling inhibited inflammatory gene expression in macrophages.^20^, ^21^ Moreover, recently, we found that EPO promoted macrophage phagocytosis of dying cells and the progression of SLE was ameliorated by exogenous EPO treatment.^22^ In addition, accumulated studies had also shown that EPO can be cytoprotective following tissue injury, indicating that EPO treatment has pronounced anti‐inflammatory and antiapoptotic capacities.^23^, ^24^ Therefore, EPO might be a candidate drug for SLE therapy, but the long‐term use of EPO has been shown to raise the risk of adverse side effects, such as thromboembolism and hypertension.^25^

Fortunately, recent studies have shown that EPO exerted its anti‐inflammatory and cytoprotective functions via the EPOR‐CD131 complex. In contrast, the haematopoietic function of EPO is considered to act through the high‐affinity homodimer of EPOR subunit.^26^, ^27^ Moreover, EPO derivatives, which only activate the protective EPOR‐CD131 complex but not the erythropoiesis EPOR homodimer, have been developed.^28^, ^29^ More recently, an EPO‐derived helix‐B peptide, ARA290, which had been reported to retain the cytoprotective properties but lack the erythropoietic activity was reported. Compared to other EPO analogs, ARA290 is a small synthetic peptide. Although ARA290 possesses a pretty short plasma half‐life, it can still exert biological functions in vivo.^30^, ^31^, ^32^ Patel et al^33^ showed that ARA290 raises the phosphorylation of survival pathway Art and inhibits the activation of pro‐inflammatory glycogen synthase kinase‐3 and NF‐κB. Furthermore, ARA290 had been reported to show protective effect for several clinical diseases, such as type II diabetes, small fibre neuropathy, rheumatoid arthritis and renal ischaemia‐reperfusion.^34^, ^35^, ^36^ However, the effect and potential mechanisms of ARA290 on SLE remain unknown so this study here sought to investigate the effects of ARA290 in SLE model.

## 2 MATERIALS AND METHODS

### 2.1 Mice and SLE induction

MRL/*lpr* mice were a kind gift from Dr. Yang, Institute of Immunology, Army Medical University, Chongqing, China, and C57BL/6 mice were purchased from The Vital River (Beijing, China). Husbandry environmental conditions consisted of 12‐hours light, 12‐hours darkness, and ambient temperature of 20°C and relative humidity of 30%‐60% at the Third Military Medical University (Chongqing, China). The animal experiments were approved by the Animal Ethics Committee of the Third Military Medical University and were performed according to the international and Chinese guidelines of animal research.

For pristane‐induced SLE model, female C57BL/6 mice, at 10‐12 weeks of age, were given a single intraperitoneal injection of 0.5 mL of pristane (Sigma‐Aldrich) to induce SLE‐like autoimmunity. At the end of the indicated experimental period, animals were killed and specimens including blood, spleen, thymus and kidney were collected.

### 2.2 ARA290 intervention

For therapy, MRL/*lpr* mice and C57BL/6 mice received intraperitoneal injection of ARA290 (peptide biochemical GmbH, Hangzhou, China; endotoxin‐free with N90% purity; 500 μg/kg/d) once daily for 3 months (6 mice/group). And the same volume of PBS was given to the control group (6 mice/group). The dosage of ARA290 was chosen according to our previous study.^34^, ^37^

### 2.3 Body and organ weight measurement

The mice were individually weighed weekly. At the end of the indicated experimental period, they were killed by cervical dislocation, and weight of organs such as spleen, kidney and lymph nodes was measured as well.

### 2.4 Histological assessment

For the assessment of renal histopathology, the mice were killed after the indicated experimental period. Mice were deeply anaesthetized with pentobarbital sodium (50 mg/kg; Boster, Wuhan, China) and were perfused with 4°C PBS and then with 4% 4°C paraformaldehyde. Kidneys were post‐fixed in 4% 4°C paraformaldehyde overnight and then cut into 2 equally long segments, embedded in paraffin. Paraffin blocks were sectioned (7 μm), and slides were stained with haematoxylin and eosin.

For immunohistochemistry staining, the paraffin‐embedded tissue blocks were cut into 7 μm sections. After dewaxing, sections were boiled (in a 600‐W microwave oven) for 15 minutes in citrate buffer (2.1 g sodium citrate/L, pH 6). The sections were cooled to room temperature, and endogenous peroxidase was inhibited with 1% hydrogen peroxidase (H2O2) in methanol for 15 minutes. To block non‐specific binding of immunoglobulins, sections were incubated with 3% albumin bovine V. Thereafter, the sections were incubated with primary antibodies overnight at 4°C. The following antibodies were used: F4/80 (BM8, ab16911; Abcam, Inc.), C3 (11H9, ab11862; Abcam, Inc.) and goat antimouse IgG antibody (ab97033; Abcam, Inc.). After washing, the sections were incubated with corresponding secondary antibodies for 30 minutes. Subsequently, the Vecta‐stain ABC Kit (Vector Laboratories, San Diego, CA, USA) was used for the avidin‐biotin complex method according to the manufacturer's protocol. Peroxidase activity was visualized with a DAB Elite Kit (K3465, DAKO, Copenhagen, Denmark). The sections were lightly counterstained with haematoxylin and dehydrated through an ethanol series to xylene and mounted.

TUNEL staining (Derma TACS, Trevigen, Inc., Gaithersburg, MD, USA) was performed to identify apoptotic cells in sections from the kidneys according to the manufacturer's protocol.

### 2.5 ELISA and biochemical parameters

Mouse anti‐dsDNA Abs Kit (Alpha Diagnostic, San Antonio, TX) and antinuclear Abs ELISA Kits (Alpha Diagnostic, San Antonio, TX) were used to detect the serologic titres of anti‐dsDNA, and antinuclear Abs, respectively. Murine serum was prepared at a 1:100 dilution in PBS, and 100 μL of the diluted serum was added to the 96‐well ELISA plates according to the manufacturer's instructions. The absorbance at 450 nm was measured with a Paradigm Multi‐Mode Plate Reader (Beckman Coulter, Fullerton, CA, USA).

The level of haemoglobin and the concentration of red blood cells in mice peripheral blood were measured in Southwest hospital (Chongqing, China).

For the assessment of urine, mice were individually kept in sterilized metabolic cages and urine samples were pooled over 24 hours. The total urinary albumin was determined with an Albumin Assay Kit (Jiancheng, Nanjing). The total protein in uria was determined by Bradford method with BCA Assay Kit (Beyotime Biotechnology, Shanghai, China). The concentrations of creatinine and urea in the urine and serum samples were measured with the Creatinine Assay Kit and Urea Assay Kit (Jiancheng, Nanjing) according to the manufacturer's instructions.

### 2.6 Flow cytometry

Flow cytometry was applied with a Mouse Inflammation CBA Kit (BD Pharmingen), including IL‐6, IL‐10, MCP‐1, IFN‐γ, TNF‐α, IL‐12p70 in serum according to the manufacturer's instructions. Flow data were collected with Cell Quest Software and analysed with FlowJo software for Windows (Treestar, Inc.)

For immunostaining and flow cytometry of bone marrow haematopoietic stem cells (HSCs) and haematopoietic progenitor cells (HPCs), bone marrow cells were flushed from femurs and stained with lineage antibodies, including CD5, CD8α, B220, CD11b, GR‐1 and Ly76, and stem cell markers, ckit, Scan1, CD34, FLT3 and FcgR. Phenotypic populations were defined as long‐term HSCs (LT‐HSCs) (Lin^−^/ckit^+^/Sca1^+^/Flt3^−^/CD34^−^), short‐term HSCs (ST‐HSCs) (Lin^−^/ckit^+^/Sca1^+^/Flt3^−^/CD34^+^), multipotent progenitors (MPPs) (Lin^−^/ckit^+^/Sca1^+^/Flt3^+^/CD34^−^), common myeloid progenitors (CMPs) (Lin^−^/ckit^+^/Sca1^+^/CD34^+^/FcγR^med^), granulocyte‐macrophage progenitors (GMPs) (Lin^−^/ckit^+^/Sca1^+^/CD34^+^/FcγR^hi^), and megakaryocyte‐erythroid progenitors (MEPs) (Lin^−^/ckit^+^/Sca1^+^/CD34^−^/FcγR^lo^). Dead cells were excluded by PI selection. Antibodies were purchased from BioLegend. Negative controls were set by cells without any antibody staining. Flow cytometry was performed on a BD FACSCanto II, and the data were analysed with FlowJo software (TreeStar).

### 2.7 Macrophage RAW 264.7 culture and intervention

The immortalized murine macrophage cell line RAW 264.7 was grown in complete Dulbecco's modified Eagle's medium (DMEM) (Gibco, Grand Island, NY) containing penicillin (100 U/mL), streptomycin (100 U/mL) and 10% foetal calf serum at 37°C and 5% CO_2_ **.** To confirm effects of ARA290 on inflammatory response, 10^6^ RAW 264.7 cells were seeded into 6‐well cell culture plates in triplicates and cultured overnight. Then, cells were washed twice with PBS, and then, ARA290 was added to culture with or without 1 mg/mL LPS overnight. Further, the mRNA levels of inflammatory cytokine were measured by semi‐quantify PCR. The primers are as follows: TNF‐α (sense, TGA TCG GTC CCA ACA AGG A; antisense, TGC TTG GTG GTT TGC TAC GA), iNOS (sense, TCT GTG CCT TTG CTC ATG ACA; antisense, TGC TTC GAA CAT CGA ACG TC) and β‐actin (sense, CCG TCT TCC CCT CCA TCG T; antisense, ATC GTC CCA GTT GGT TAC AAT GC).

### 2.8 Generation of apoptotic cells and the in vitro phagocytosis assays

To generate apoptotic Jurkat cells, cells were cultured in RPMI without FCS, and apoptosis was induced with 0.5 μg/mL staurosporine (Sigma‐Aldrich) for 3 hour. Afterwards, cells were washed 3 times with PBS and resuspended in 10% FCS in RPMI. Staurosporine treatment yielded a population with 90% apoptotic cells.

Before being fed to macrophages, apoptotic cells were labelled with pHrodo™ Green (pHrodo, Molecular Probes), a pH‐sensitive phagocytosis‐dependent indicator that requires no wash steps or quenchers, according to the manufacturer's instructions. Macrophages were incubated with pHrodo‐labelled apoptotic cells at a ratio of 1:5 (macrophages: apoptosis cells) and cultured at 37°C for 60 minutes in RPMI supplemented with 10% FBS. Meantime, macrophages were pretreated with ARA290 before co‐incubation with pHrodo‐labelled apoptotic cells. After incubation, cells were resuspended and stained with fluorescence‐labelled anti‐F4/80 (BM8; Sungene Biotech) and anti‐CD11b (M1/70; Sungene Biotech). Then, they were analysed by flow cytometry and the proportion of macrophages containing ingested apoptotic cells was determined.

### 2.9 Statistical analysis

Data were calculated with a statistical software package (GraphPad Prism 5.0 for Windows). Data were presented as the means ± SEM, and the group mean values were analysed by the two‐tailed unpaired Student's *t* test. For all statistical analyses, statistical significance is indicated by a single asterisk (*P *<* *.05), 2 asterisks (*P *<* *.01) or 3 asterisks (*P *<* *.001).

## 3 RESULTS

### 3.1 ARA290 treatment Ameliorates Lupuslike symptoms in pristane‐induced SLE mice

**Figure 1:**
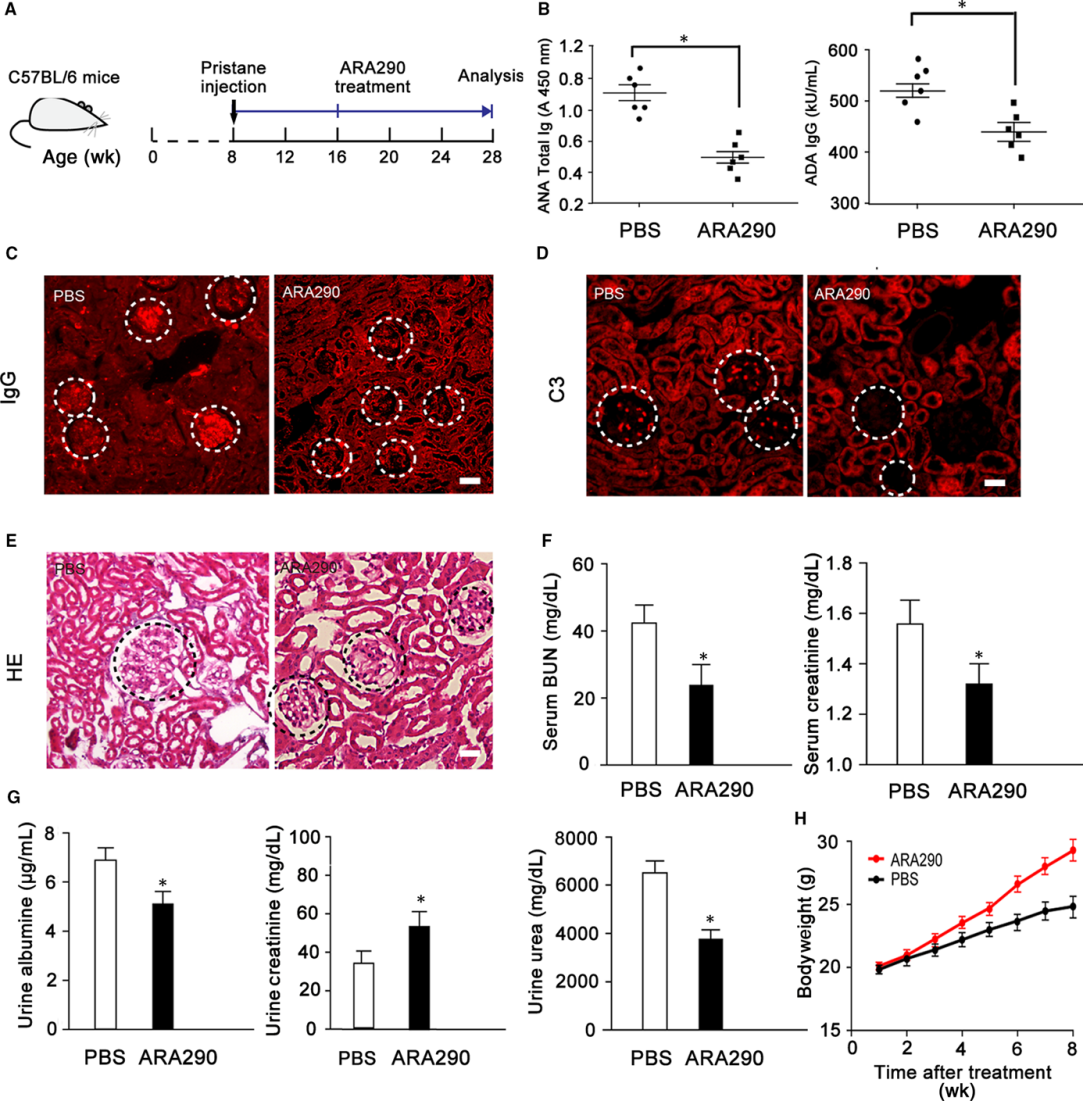
ARA290 treatment Ameliorates Lupuslike symptoms in pristane‐induced SLE mice (A) Eight‐week‐old female SLE mice were orally administered PBS (n = 6) or ARA290 (n = 6) 3 times a week until the end of study period, and the mice were killed, and kidney was taken for HE and immunohistochemical staining. (B) The serum levels of total ANAs and the IgG anti‐dsDNA antibody following ARA290 treatment were measured. (C‐E) Representative micrographs show that ARA290 treatment greatly reduces accumulation of IgG and C3 and histological score for glomerulus (HE) in kidney of SLE mice. (F‐G) The serum levels of BUN, creatine and the urine levels of albumine, creatine and urea were detected at the end of study period. (H) Bodyweight was measured once a week during ARA290 treatment. Scale bars represent 30 μm. Graphs show the mean ± S.E.M. *P <* *.05 compared to their respective vehicle control. ANA, antinuclear antibody; dsDNA, double‐stranded DNA; BUN, blood urea nitrogen. Representative data from 2 independent experiments are shown

We first investigated whether ARA290 altered the development of lupuslike autoimmune disease with the pristane‐induced murine lupus model, which is characterized by the production of autoantibodies and development of glomerulonephritis, resembling the key features of human SLE. Moreover, pristane also leads to persistent apoptosis of immature monocytes in ectopic lymphoid tissue that forms in response to the hydrocarbon. Eight weeks following pristane injection, ARA290 was given to this SLE mice once daily for 12 weeks (Figure 1A).

One of the most reliable criteria for SLE is an increase in antinuclear antigen autoantibodies in the serum.^38^ A significant decrease in the serum concentrations of anti‐dsDNA antibodies (ADA) and antinuclear antibodies (ANA) was observed in ARA290‐treated SLE mice compared to the control mice (Figure 1B). Another hallmark of SLE is the deposition of autoantibodies as immune complexes in the kidney, leading to inflammation and destruction of the glomeruli. Following ARA290 treatment, the deposition of IgG and complement C3 in glomerulus was significantly reduced than in control mice (Figure 1C, D). Furthermore, reduced glomerular size and cellularity were observed in kidney sections from ARA290‐treated mice than from their vehicle counterparts (Figure 1E). In line with the pathological changes in the kidney, the levels of creatinine and blood urea nitrogen in serum were greatly decreased following ARA290 treatment compared to the control (Figure 1F). Accordingly, reduced levels of diuresis and urine albumin and increased levels of urine creatinine were found in the ARA290‐treated mice compared to that of the vehicle control group (Figure 1G). In addition, the bodyweight of the ARA290‐treated SLE mice was greatly higher than that of the control SLE mice, indicating a better outcome. Collectively, these data show that ARA290 treatment produces improvements in lupuslike symptoms in pristane‐induced SLE mice.

### 3.2 ARA290 treatment suppressed inflammatory response in pristane‐induced SLE mice

**Figure 2:**
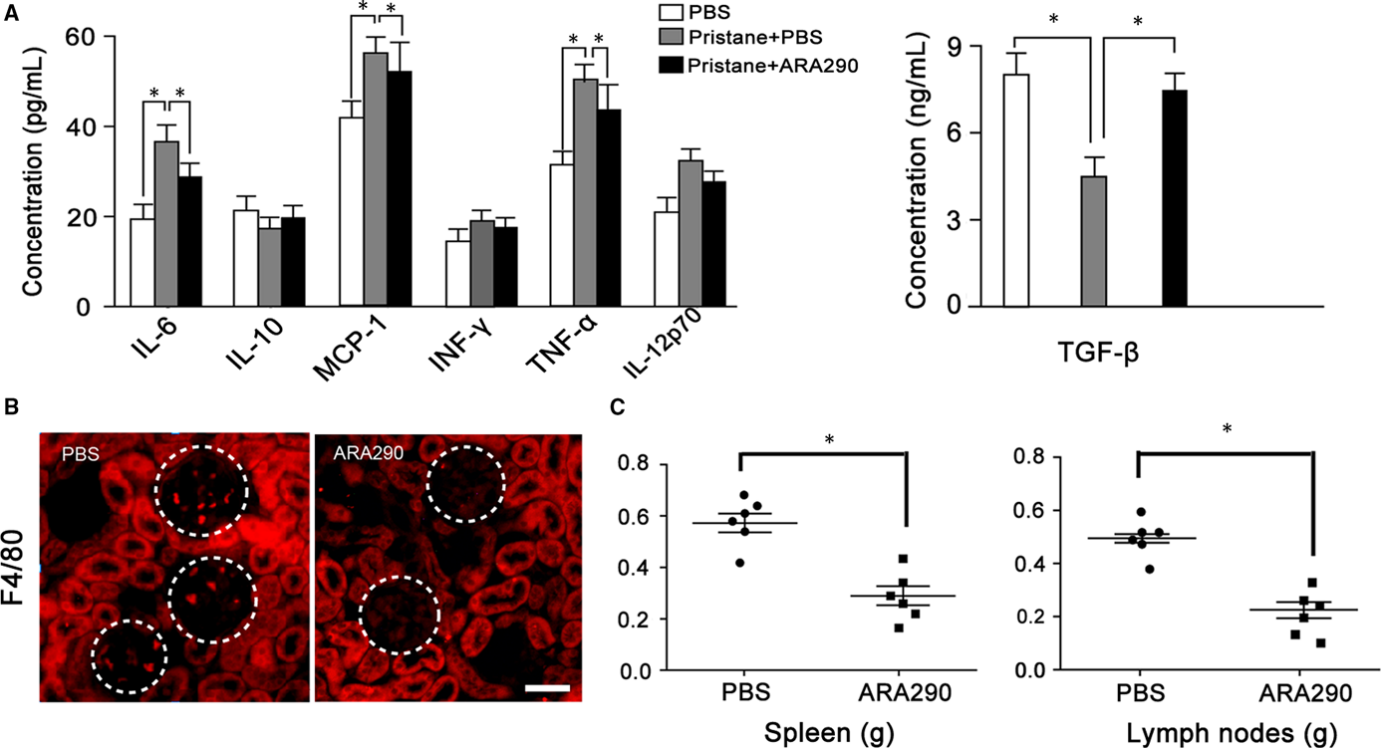
ARA290 treatment suppressed inflammatory response in pristane‐induced SLE mice. (A) The levels of IL‐6, IL‐10, MCP‐1, IFN‐γ, TNF‐α, IL‐12p70 and TGF‐β in the serum following treatment described in Figure 1 were detected (n = 6). (B) The inflammatory macrophage F4/80 infiltration was significantly suppressed by ARA 290 intervention compared to PBS control. (C) The spleen and lymph node weights were measured following ARA290 treating in SLE mice (n = 6). Scale bars represent 30 μm. *: *P *<* *.05 compared to their respective vehicle control. Representative data from 2 independent experiments are shown

Inflammation plays a vital role in SLE development. In SLE, IL‐6, MCP‐1, IFN‐γ and TNF‐α promotes the progression of this disease.^39^, ^40^, ^41^, ^42^ On the contrary, IL‐10 and TGF‐β are anti‐inflammatory cytokines, which are related to SLE recovery.^14^, ^43^ As shown in Figure 2A, ARA290 treatment significantly decreased the serum levels of IL‐6, MCP‐1 and TNF‐α, and increased the serum levels of TGF‐β, suggesting an inflammation suppressing role of ARA290 in SLE.

Moreover, macrophages are reported to contribute to the pathogenesis of SLE and further studies have demonstrated that macrophage infiltration to kidney is associated with poor outcome of SLE. Then, we further investigated the macrophage accumulation in SLE mouse kidney following ARA290 treatment. As shown in Figure 2B, the infiltration of F4/80^+^ macrophages into the kidney was much less evident in ARA290 group compared to control group.

In addition, the enlargement of spleen and lymph nodes is related with active inflammatory response and regarded as clinical markers in lupus patients. In current investigation, we found the weight of the spleen and lymph nodes of SLE mice was significant reduced following ARA290 treatment compared to that of the control mice (Figure 2C).

Together, these data demonstrate that ARA290 treatment reduced the inflammatory response in pristane‐induced SLE mice.

### 3.3 ARA290 Inhibited Inflammatory Macrophage Activation but Promoted its Phagocytic Activity

Accumulated evidences suggested a more active role of macrophages in mediating tissue inflammation and injury in SLE, including high inflammatory cytokine production and defective phagocytic capacity.^44^, ^45^ Then, we next investigated ARA290 effects on macrophage.

**Figure 3:**
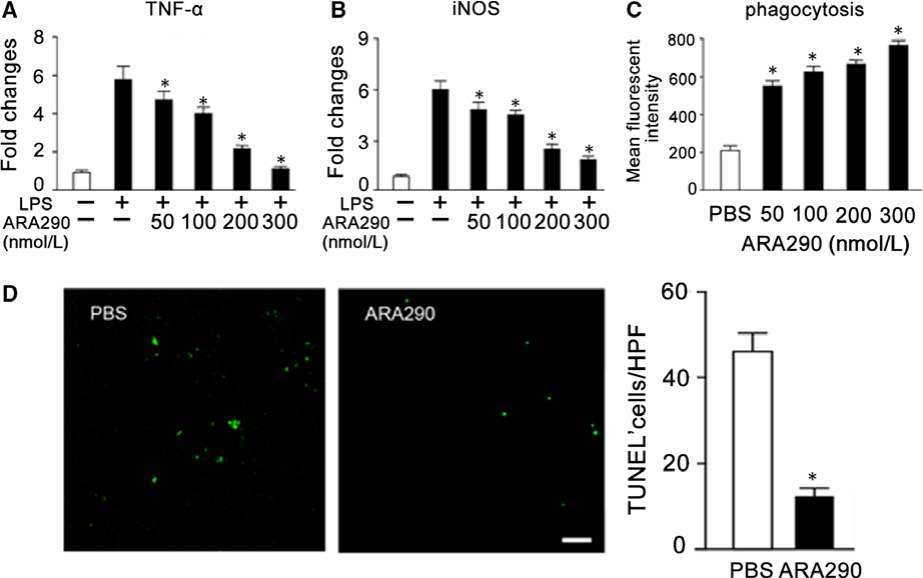
ARA290 Inhibited Inflammatory Macrophage Activation but Promoted its Phagocytic Activity in vitro. (A‐B) RAW 264.7 cells were treated with 1 mg/mL LPS followed by ARA290 (n = 4) or PBS control (n = 4) intervention with various doses for 4 h. The mRNA levels of TNF‐α and iNOS were measured. (C) Following ARA290 pretreatment, macrophages were incubated with pHrodo‐labelled apoptotic cells for 1 h and then analysed by flow cytometry (n = 4). (D) PBS or ARA 290 (500 μg/kg/d) was given to pristane‐induced SLE mice from week 16 to week 28. And the apoptotic cell accumulation in kidney following ARA290 treatment was detected by TUNEL staining (n = 6). Scale bars represent 30 μm.*: *P *<* *.05 compared to their respective vehicle control. Representative data from 2 independent experiments are shown

We first studied the ARA290 effects on inflammatory macrophage activation, and LPS was applied to stimulate macrophages. As shown in Figure 3A, B, increased expression of TNF‐α and iNOS was detected following LPS stimulation, indicating an inflammatory macrophage activation. And ARA290 dose‐dependently suppressed LPS‐induced expression of TNF‐α and iNOS (Figure 3A, B).

The defective apoptotic cell clearance has been linked to autoimmune disease, such as SLE, and the accumulation of apoptotic cells and decreased phagocytotic ability of macrophages in SLE have been reported.^46^, ^47^, ^48^ Therefore, we further detected the impact of ARA290 on apoptotic cell clearance by macrophages in vitro by flow cytometry. As shown in Figure 3C, ARA290 dose‐dependently promoted macrophage phagocytosis of apoptotic cells. In line with this in vitro observation, we also found that the accumulation of apoptotic cells in kidney of pristane‐induced SLE mice was greatly decreased by ARA290 (Figure 3D).

Collectively, these data show that ARA290 suppressed macrophage inflammatory activation and promoted macrophage phagocytosis of apoptotic cells.

### 3.4 ARA290 did not Stimulate Haematopoiesis during SLE treatment

**Figure 4:**
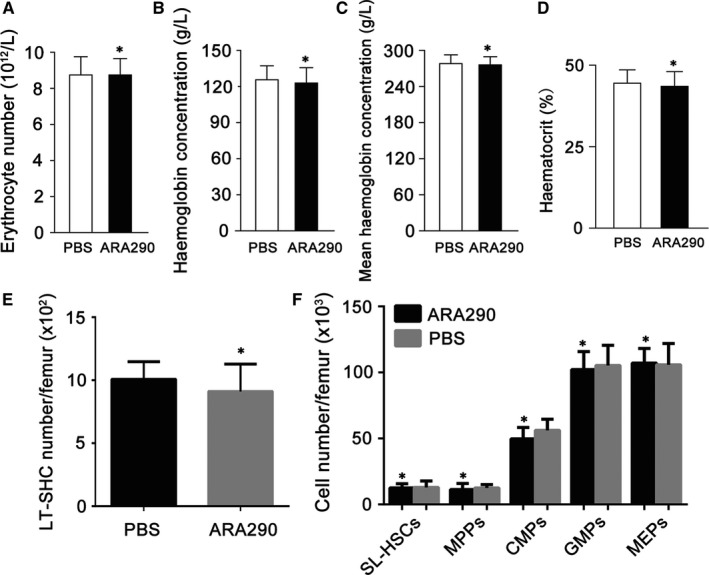
ARA 290 did not Stimulate Haematopoiesis during SLE treatment in mice. (A‐D) PBS or ARA 290 (500/kg/d) was given to SLE mice (n = 6) for 12 weeks, and mice were killed to take peripheral blood for the measurement of erythrocyte cell number, haemoglobin concentration, mean haemoglobin concentration and haematocrit. Compared to PBS group, ARA 290 treatment did not increase erythrocyte cell number, haemoglobin concentration or haematocrit. (E‐F) Wild‐type mice were treated with ARA290 (500 μg/kg/d, n = 4) or PBS (n = 4) for 28 days, and then, mice were killed to take the bone marrow for the measurement of haematopoietic stem and progenitor cells. LT‐HSCs, long‐term haematopoietic stem cells; SL‐HSCs, short‐term haematopoietic stem cells, MPPs, multipotent progenitor cells; CMPs, common myeloid progenitors; GMPs, granulocyte‐monocyte progenitors; MEPs, megakaryocyte‐erythroid progenitors. Representative data from 2 independent experiments are shown. *: *P *<* *.05 compared to their respective vehicle control

In addition, we detected whether ARA290 stimulates haematopoiesis during SLE intervention. For pristane‐induced SLE mice treatment, ARA290 was given once daily for 12 weeks. Compared to the vehicle control group, ARA290 administration did not increase the numbers of peripheral blood cell, the percentage of haemoglobin levels and haematocrit (Figure 4A‐D), implying that ARA290 did not stimulate haematopoiesis during SLE treatment.

To further investigate the effects of ARA290 on bone marrow HSCs and HPCs, C57BL/6 mice were treated with ARA290 (500 μg/kg/d, n = 4) or PBS (n = 4) for 28 days, and then, mice were killed to take the bone marrow for the measurement of HSCs and HPCs. Using flow cytometry and immunostaining of HSCs and HPCs, we found that the absolute numbers of long‐term (LT) HSCs, short‐term (ST) HSCs, and multipotent progenitor cells (MPPs), common myeloid progenitors (CMPs), granulocyte‐monocyte progenitors (GMPs) and megakaryocyte‐erythroid progenitors (MEPs) were comparable between the treated and control groups (Figure 4E, F), indicating that the application of ARA290 has no significant effects on the numbers of bone marrow haematopoietic stem and progenitor cells.

### 3.5 ARA290 treatment Ameliorates Lupuslike symptoms in MRL/*lpr* SLE mice

**Figure 5:**
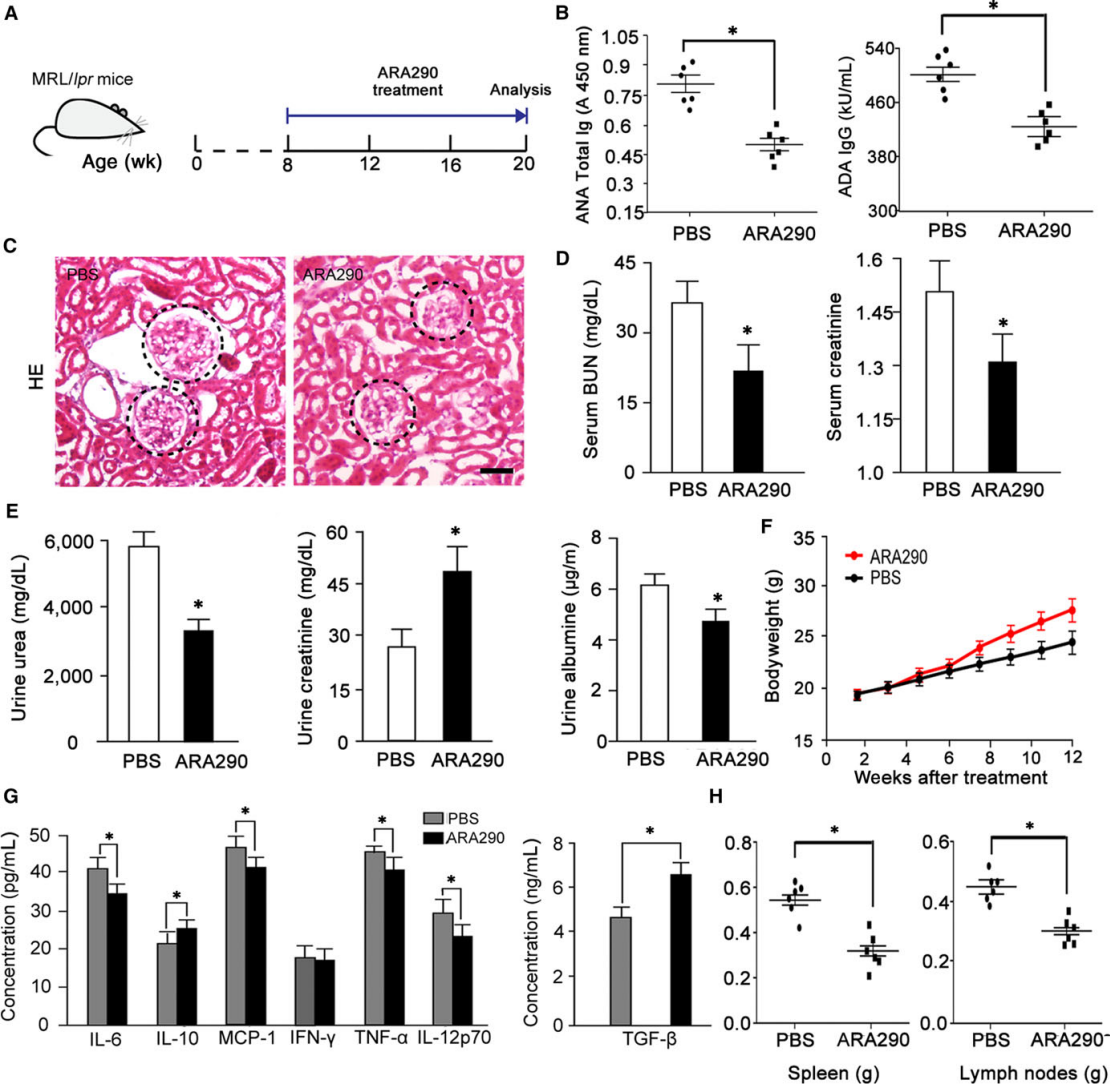
ARA290 treatment Ameliorates Lupuslike symptoms in MRL/*lpr* Mice. (A‐H), ARA290 (500 μg/kg/d**,** 3 times/wk, n = 6) or PBS (n = 6) treatment of MRL/*lpr* mice. Following treatment, serum ANA and ADA concentrations (B), Histological score for glomerular tuft according to H&E staining (C), kidney function (D, E), bodyweight (F), serum cytokine concentrations (G), and the spleen and lymph node weights (H) were measured. Scale bars represent 30 μm. *: *P *<* *.05 compared to their respective vehicle control

To verify the effect of ARA290 on SLE, we further analysed the effect of ARA290 in the MRL/*lpr* SLE mice. Being a classic SLE animal model, female MRL/*lpr* mice will spontaneously develop autoimmune syndromes characterized by lupus nephritis, haematological changes, massive lymphadenopathy, splenomegaly and autoantibody formation, among which lupus nephritis is the key factor that leads to death.^49^ ARA290 was given to 8‐week female MRL/*lpr* mice once daily for 12 weeks (Figure 5A). Like in pristane‐induced SLE mice, ARA290 treatment greatly reduced the serum levels of ADA and ANA (Figure 5B), decreased the glomerular size and cellularity in kidney (Figure 5C), improved the kidney functions (Figure 5D, E) and improved the bodyweight (Figure 5F) in the MRL/*lpr* SLE mice. In addition, ARA290 treatment significantly decreased the serum levels of IL‐6, MCP‐1, TNF‐α and IL‐12p70, and increased the serum levels of TGF‐β and IL‐10 in MRL/*lpr* SLE mice, suggesting an inflammation suppressing role of ARA290 in SLE (Figure 5G). Furthermore, the enlargement of spleen and lymph nodes was reduced by ARA290 as well (Figure 5H).

Collectively, these data show that ARA290 treatment produces improvements in lupuslike symptoms and suppressed inflammation in the MRL/*lpr* SLE mice.

## 4 DISCUSSION

Here, we have explored the effects of ARA290 on SLE mice. We found that the administration of ARA290 to pristane‐induced SLE mice and MRL/lpr mice significantly suppressed the level of serum ANAs and anti‐dsDNA autoantibodies, reduced the kidney deposition of IgG and C3, and ameliorated the nephritis symptoms. Moreover, the serum concentrations of pro‐inflammatory cytokine, such as IL‐6, MCP‐1 and TNF‐α, were reduced, but the anti‐inflammatory cytokine TGF‐β was induced by ARA290 in SLE mice. In addition, ARA290 decreased the number of apoptotic cells in kidney of SLE mice. In vitro experiment revealed that ARA290 inhibited the inflammatory activation of macrophages and promoted the phagocytotic function of macrophages to apoptotic cells. Finally, ARA290 did not induce haematopoiesis during treatment. These data demonstrate that ARA290 ameliorated SLE, which at least could be partly due to its anti‐inflammatory and apoptotic cell clearance promoting effects, without stimulating haematopoiesis, suggesting that ARA290 could be a hopeful candidate for SLE treatment.

The increased evidence suggested a close link between pro‐inflammatory cytokines and SLE. Specifically, up‐regulated pro‐inflammatory cytokines caused an immune dysregulation and tissue damage.^50^, ^51^ Fox example, studies have revealed that elevated serum levels of TNF‐α are positively associated with disease activity as well as renal involvement in patients with SLE.^10^, ^52^ Moreover, IL‐6 can be hoisted in the urine of lupus nephritis patients, with higher levels correlating with active renal inflammation and pathology.^53^, ^54^ In addition, monocyte chemoattractant protein (MCP‐1) is implicated in the activation of inflammatory cells and has been suggested to affect the progression of lupus nephritis (LN).^55^, ^56^ Macrophages are the main origin of these inflammatory cytokines in SLE. In the current study, our in vivo and in vitro studies showed that ARA290 ameliorated inflammatory response in SLE mice and, notably, suppressed the expression of TNF‐α, IL‐6, iNOS and MCP‐1 in macrophages, which suggested that ARA290 might alleviated the symptoms of SLE via regulating macrophage inflammatory response; however, the definite mechanism between ARA290 and macrophage inflammatory response needs further investigation.

Our study also provides forceful evidence that ARA290 significantly promote the phagocytic ability of macrophages in vitro and in vivo. SLE is associated with the deposition of apoptotic cells in vivo.^45^ Defects in apoptotic cell clearance may result in secondary necrosis and lead to autoimmunity.^45^, ^57^ Research had reported that macrophages are the main phagocytic cells. In 1980s, studies had shown that the defect phagocytic function of macrophages would lead to abnormal accumulation of apoptotic debris, considered to be SLE macrophages in vivo abnormalities.^58^ Our previous study has shown that EPO promotes macrophage phagocytosis of apoptotic cells.^22^ In line with this, here we found that the EPO‐derived ARA290 peptide also elevated the phagocytotic activity of macrophage to apoptotic cells in vitro. Furthermore, the apoptotic cell accumulation in vivo was also reduced following ARA290 treatment in SLE. Therefore, the therapeutic effects of ARA290 on SLE could be partly due to its activity to promote apoptotic cell clearance.

In conclusion, our investigation here demonstrates that ARA290 ameliorated SLE, which at least could be partly due to its anti‐inflammatory and apoptotic cell clearance promoting effects, without stimulating haematopoiesis, suggesting that ARA290 could be a hopeful candidate for SLE treatment.

## CONFLICT OF INTEREST

The authors declare that they have no conflict of interest.

## AUTHOR CONTRIBUTION

ZZ and BL provided the idea and conceived and designed the experiments. WZ, BL, YL and TL performed the experiments and analysed the data. B.H., WZ and ZZ wrote the manuscript, ZZ and BL supervised the study.
